# Evaluating a multi-component intervention to reduce and break up office workers’ sitting with sit-stand desks using the APEASE criteria

**DOI:** 10.1186/s12889-022-12794-w

**Published:** 2022-03-07

**Authors:** Marsha L. Brierley, Lindsey R. Smith, Daniel P. Bailey, Samson O. Ojo, David J. Hewson, Sofie A. Every, Taylor A. Staines, Angel M. Chater

**Affiliations:** 1 Institute for Sport and Physical Activity Research, School of Sport Science and Physical Activity, University of Bedfordshire, Polhill Avenue, Bedford, MK41 9EA UK; 2 Division of Sport, Health and Exercise Sciences, Department of Life Sciences, Brunel University London, Uxbridge, UB8 3PH UK; 3 Centre for Physical Activity in Health and Disease, Brunel University London, Kingston Lane, Uxbridge, UB8 3PH UK; 4 Institute for Health Research, University of Bedfordshire, University Square, Luton, LU1 3JU UK; 5 Quality Improvement, Northampton General Hospital NHS Trust, Cliftonville, Northampton, Northamptonshire, NN1 5BD UK; 6 Centre for Behaviour Change, University College London, 1-19 Torrington Place, London, WC1E 7HB UK

**Keywords:** Sedentary, Behaviour change, Intervention, Office worker, Feasibility, Acceptability, Apease

## Abstract

**Objective:**

Sedentary workplace interventions have had success in reducing excessive sitting time in office workers, but barriers to implementation and uptake remain. This study formally assessed a theory-derived, sit-stand desk intervention using the APEASE (Acceptability, Practicability, Effectiveness, Affordability, Side-effects, Equity) criteria.

**Methods:**

Thirteen adults (eight female, mean age 38 ± 10 years) from the treatment arm of a sedentary behaviour intervention participated in semi-structured interviews. Thematic codes were inductively assigned to data items followed by deductive charting using the APEASE criteria.

**Results:**

The intervention was highly acceptable, practicable, safe to deploy, and helped workers reduce workplace sitting time, though individual preferences and workload mediated engagement. Affordability of sit-stand desks and Equity of access were potential barriers to uptake.

**Conclusions:**

Through the lens of the APEASE criteria, this theory-derived, multi-component sit-stand desk intervention showed acceptability, practicability and effectiveness in reducing and breaking up sedentary time at work with minimal side effects. Using this approach with further tailoring and personalisation may help workers achieve greater reductions in workplace sitting, though affordability and equity should be considered further.

**Supplementary Information:**

The online version contains supplementary material available at 10.1186/s12889-022-12794-w.

## Introduction

Engaging in high volumes of daily sedentary time (e.g. ≥ 6–8 h per day) [1] is associated with increased risk of developing type 2 diabetes, cardiovascular disease, some cancers and premature death, often independent of physical activity [2–5]. Engaging in at least 60–75 min of moderate physical activity per day may be needed to offset the adverse health effects of excessive sitting time [6]. In the workplace, sedentary behaviour has been operationally defined as prolonged sitting [7] which can be considered as any sitting bout lasting more than 30 min [8]. Office workers typically sit for the majority of their workday and accrue the majority of their sitting time in prolonged bouts [9–12]. For example, in a recent study of Welsh office workers, 69% of the workday was spent seated and 64% of that time was accumulated in bouts over 30 min [12].

The ability to reach a large number of people in a setting that is synonymous with prolonged sitting presents an opportunity to improve public health via workplace interventions [7]. However, intervention success may depend on how much and to what extent workers adopt sitting time recommendations. Workplace interventions have significantly reduced workplace sitting by 30 to 120 min [13]. However, there may be remaining barriers that affect their success, such as social norms that reinforce prolonged sitting (social/organisational level barrier), office layouts that discourage movement (environmental level barrier), and forgetting to use provided resources like sit-stand desks (individual level barrier) [14, 15]. Understanding how and why intervention resources are used by participants will help researchers better understand acceptability and feasibility issues to enhance the effectiveness of future interventions.

Qualitative studies on single-component sit-stand desk interventions show positive overall acceptability and feasibility [15–18]. This is also the case for dual-component interventions that pair sit-stand desks with an initial educational/training session [19–21] or ongoing motivational support [22]. With regards to multi-component interventions, less is known about the participant experience. Hadgraft et al. [23] found that an intervention using a combination of environmental (sit-stand desks), individual (one-to-one health coaching), and organisational level support (management emails) was perceived as positively impacting participant health and well-being with few negative effects on productivity. Participants also perceived a shift towards a standing work culture [14]. However, despite having sit-stand desks and additional support, sitting and standing were often task and situation dependent, and workstation design could be a barrier to use [14]. Call centre employees in a multi-component intervention found the strategies of education/training, regular email support, and behavioural feedback acceptable [24]. However, sitting timers (for goal setting and self-monitoring) were not perceived as practicable at work and health champions were perceived as ineffective in the call centre setting [24]. Furthermore, time pressure, social norms, and few physical and/or social opportunities remained barriers to reducing workplace sitting time [24]. There is a need to further assess the feasibility of multi-component intervention strategies in other occupational sub-groups to provide a wider evidence base for understanding what works and why.

Current recommendations suggest initially replacing two hours of a typically seated 8-h workday with standing or light activity in addition to frequent posture changes [7]. In previous studies, sit-stand desk interventions have typically led to reductions of 30 – 120 min in workday sedentary time in the short term (3 months or less) [13, 25, 26]. Longer term changes are less known, but a recent review found that two sit-stand desk interventions with longer term follow-ups between 3 – 12 months had an average reduction of 57 min per workday [13]. Furthermore, sedentary workplace interventions show promise for improving health outcomes [27]. Understanding the participant experience will help researchers to curate and design resources to help participants reach and sustain recommended behavioural targets for workplace sitting to benefit health outcomes, such as cardiometabolic disease risk and psychological wellbeing.

The Behaviour Change Wheel (BCW) [28, 29] provides a framework to assess and address stakeholder needs for effective behaviour change interventions. The BCW approach incorporates the Behaviour Change Technique Taxonomy v1, a theory-based taxonomy of 93 individual techniques for behaviour change [30]. The BCW also incorporates the APEASE criteria to assess Acceptability, Practicability, Effectiveness, Affordability, Side-effects, and Equity, iteratively in all stages of intervention design, delivery and evaluation [29, 31]. In the initial stages of intervention development, APEASE aids researchers to systematically make decisions about which behaviour change techniques (BCTs), intervention functions, and policy categories to include [32, 33]. In the later stages, APEASE can be used to assess intervention acceptability and feasibility in a thorough and methodical way. The primary aim of this study was to use the APEASE criteria to assess the experiences of those who participated in a multi-component sedentary workplace intervention involving sit-stand desks. This evaluation will help inform the design of future sedentary workplace interventions.

## Methods

### Study setting and design

This qualitative study consisted of semi-structured interviews with participants from a multi-component, sit-stand desk pilot intervention [32, 34]. Twenty desk-based university and local-authority employees from the East of England assigned to the treatment arm of the intervention were invited via email to participate in post-intervention interviews. Ethical approval for the post-intervention interview study was granted from the University of Bedfordshire Institute for Sport and Physical Activity Research Ethics Committee (2018ISPAR014) and was performed in accordance with the Declaration of Helsinki for research involving human participants. Interview participants provided written informed consent including permission for the publication of anonymised quotations. The present study follows the COnsolidated Criteria for REporting Qualitative studies (COREQ), a 32-item checklist for interviews and focus groups (see Table, Additional file 1, which shows the completed COREQ checklist) [35].

### The intervention

Employees were recruited between April–May 2018 for a pilot cluster-randomised controlled trial (prospectively registered at clinicaltrials.gov: NCT03560544). Participants were between 18–60 years old, working ≥ 0.6 full time equivalent hours, self-reporting at least 5.5 h of workplace sitting per 8-h workday (measured using a domain-specific sitting questionnaire [36]), ambulatory, and not pregnant [34]. After baseline measures, fourteen offices/clusters (averaging three people each) were randomly assigned to the intervention or control group by a researcher not involved in the study. The intervention study ran for eight weeks (July – August 2018) and consisted of environmental (sit-stand desks), individual (prompt software), and organisational level (education and motivational email support) components. Total sitting and patterns of sitting time at work were device-assessed using the thigh-worn activPAL3 tri-axial accelerometer (PAL Technologies, Glasgow, UK) at baseline and during the final week of the intervention (week 8). Cardiometabolic health outcomes were assessed at baseline and within five days post-intervention including fasted glucose and lipid profile, resting blood pressure, waist circumference, and body mass index. Questionnaires on stress [37], mood [38], wellbeing [39], and self-reported work productivity [40, 41] were administered at baseline and post-intervention. Ecological momentary assessment of productivity was measured throughout the intervention period.

#### Control group

Control group participants did not receive the intervention and were asked to maintain their usual work practices. After completion of all follow-up measures, control group participants were offered the use of the sit-stand desks for eight weeks.

### Behaviour change techniques

The intervention was developed using the BCW and BCT selection as described in full elsewhere [32, 34]. During the eight-week intervention, workers participated together in clusters of two to three people (BCT: Restructuring the social environment). Intervention participants’ regular desks were equipped with height-adjustable workstations (WorkFit-T Sit-stand Workstation; Ergotron, Inc., Saint Paul, MN, USA). These were positioned on top of their normal desk and could be raised or lowered via hand-brake levers to allow participants to change posture while working (BCTs: Behavioural practice/rehearsal, Restructuring the physical environment, Adding objects to the environment). A 5–10-min information session was held to show participants how to break up sitting by using the desks (BCT: Demonstration of the behaviour), to hand out education leaflets with evidence-based workplace sitting recommendations and tips for achieving guidelines (BCTs: Instruction on how to perform the behaviour, Credible source), and to provide information about health and emotional consequences (BCTs: Information about health consequences, Information about emotional consequences). Participants were verbally instructed to problem solve and create action plans for gradually increasing their sitting reduction over the eight weeks (BCTs: Problem solving, Action planning, Graded tasks) as well as to set bi-weekly sitting reduction goals [BCT: Goal setting (behaviour)]. Prompting software (Marinara Timer; Three Five Two, Inc., Atlanta, GA, USA) was installed on individual computers to remind participants to frequently change between sitting and standing postures and to take regular short breaks (stepping) in line with their goals (BCT: Prompts/cues). The prompting software could be used as a Pomodoro method timer (i.e., a 25-min timed focus period followed by a 5-min break, then a 15-min break after the fourth round) or with focus/break settings customised by the user. Bi-weekly emails were sent via line-managers reiterating participants’ stated goals [BCT: Social support (unspecified)].

### Interview schedule

Semi-structured interview questions were prepared that asked participants on their use of intervention resources, facilitators and barriers to participation, and perceived impacts of the intervention on physical, social, and psychological outcomes (see Table, Additional file 2, which shows the interview schedule). The interview schedule was modelled on Hadgraft et al.’s [23] study on employee perceptions of a multi-component intervention with sit-stand desks. Additional questions were adapted from prior qualitative studies on sit-stand desk use [16, 17] and walking programme engagement [42]. The questions were pilot tested on an individual not involved in the intervention who partly worked at a standing only desk. Questions were then reordered for clarity and technical jargon replaced with layperson terminology for ease of understanding (for example, ‘sedentary behaviour’ was replaced with ‘sitting’).

### Procedure

Prior to interview, participants in the current study were asked to complete an online demographic survey (Qualtrics Inc., Seattle, WA, USA) stating their age, sex, job type (‘I manage others’/’I do not manage others’), length of tenure with the organisation, hours worked per week, education level, marital/cohabitation status, exercise behaviours, health status, smoking status, and typical alcohol consumption. One-to-one interviews were performed by the lead researcher (MLB) within 1 – 4 weeks post-intervention. All interviews took place in person during work time at the participants’ place of employment. Interviews were audio-recorded in person using a Tascam DR-05 dictaphone (TEAC Corporation, Montebello, CA, USA). Conversations were transcribed verbatim (by MLB, SAE, TAS) using Express Scribe software (NCH Software, Inc., Canberra, Australia) and an Infinity IN-USB-2 foot pedal (AltoEdge Pty Ltd., Colorado, USA). Transcripts were de-identified and anonymised prior to analysis.

### Analysis

Descriptive statistics (means, standard deviations, and frequencies) were calculated for demographic data using Excel (version 16, Microsoft Corporation, Albuquerque, NM, USA).

Qualitative data was analysed using NVivo software (version 11, QSR International Pty Ltd, Melbourne, Australia). First, an inductive approach using thematic analysis was used to identify nodes and sub-themes in the data [43]. Second, a deductive approach was used to ‘chart in’ sub-themes to the APEASE criteria [44, 45]. Transcripts were initially coded line by line. Preliminary code names were assigned to these data items and iteratively developed throughout the coding process by MLB. An expert in qualitative research, the BCW, and psychology (AMC) checked and discussed all transcripts for preliminary code names and helped to deductively assign them to the APEASE criteria [28, 29, 31] through an iterative process (see Table 1). The data was discussed with LRS and DPB to refine and confirm the final interpretations [46].

**Table 1:** The APEASE criteria for assessing intervention acceptability, practicability, effectiveness, affordability, side-effects and equity [31]

Criteria	Description
Acceptability	How far is it acceptable to key stakeholders? This includes the target group, potential funders, practitioners delivering the interventions and relevant community and commercial groups.
Practicability	Can it be implemented at scale within the intended context, material and human resources? What would need to be done to ensure that the resources and personnel were in place, and is the intervention sustainable?
Effectiveness	How effective is the intervention in achieving the policy objective(s)? How far will it reach the intended target group and how large an effect will it have on those who are reached?
Affordability	How far can it be afforded when delivered at the scale intended? Can the necessary budget be found for it? Will it provide a good return on investment?
Side-effects	What are the chances that it will lead to unintended adverse or beneficial outcomes?
Equity	How far will it increase or decrease differences between advantaged and disadvantaged sectors of society?

### Data saturation

The APEASE criteria helped provide an objective means of structuring responses and monitoring data saturation. Data saturation was confirmed after discussion and agreement with a second researcher (AMC) that no new information could be identified either directly from the responses [47] or further interpreted in the form of aggregate themes from the analysis [47]. No new information was identified in the final analysis, and, therefore, it was not necessary to conduct additional interviews [48, 49].

## Results

### Participants

Thirteen employees consented to interview, with sessions lasting an average of 33 ± 6 min. The sample was 62% female (*n* = 8), average age 38 ± 10 years, with 85% having a university or higher degree (*n* = 11). Just over half of the sample was married/cohabiting (*n* = 7, 54%). Sixty-two percent of interview participants were managers (*n* = 8), and average length of tenure at the organisation was 7 ± 7 years. Participants worked an average of 34 ± 9 h per week. Just over half of the sample (*n* = 7, 54%) self-reported not meeting the recommended 150 min of moderate to vigorous physical activity per week [50]. Sixty-nine percent of participants (*n* = 9) self-rated their overall health as good, very good or excellent. Most had never smoked tobacco (*n* = 11, 85%), and 92% (*n* = 12) reported alcohol use within recommended UK guidelines (≤ 14 units per week) [51].

### APEASE

Data is narratively presented using the framework subheadings of the APEASE criteria: Acceptability, Practicability, Effectiveness, Affordability, Side-effects, and Equity. Participant quotations, identified by pseudonym, are provided to illustrate key concepts.

#### Acceptability

Overall, participants felt the intervention was highly acceptable and enjoyable:

*“It [the intervention] was a good experience.” *(P3)

The sit-stand desk was acceptable, with participants expressing a desire to keep it:

“I love it, the desk. I found it, I got on with it really well. I didn’t find it a hindrance at all.” (P12)

“I'm happy with them [the sit-stand desks], I’d like to keep it.” (P7)

The length of the intervention (eight weeks) was appropriate for getting used to breaking up sitting time and gradually increasing use of their sit-stand desk:

“It didn’t take that long to get used [to it]. It was only probably only two weeks until it became, you know, natural to be standing where, when almost the default [was] to be standing.” (P4)

“The eight weeks just made it [breaking up sitting] more automatic, I think.” (P5)

The intervention was delivered over the summer and the hot weather sometimes made standing uncomfortable:

“Maybe the fact that it [the intervention] was in summer? At some point it was just really hot, and I don’t think any of us were able to stand during that period.” (P10)

Participants made suggestions for improving the intervention:

“I’m keen to continue to stand more during my day, not just at my desk. With thinking about how I can incorporate standing during meetings and other elements of my work.” (P4)

“I think it’s putting all the tools [the sit-stand desk, the education leaflet, the prompt software, the bi-weekly emails] and packaging it all together in a nice package.” (P2)

There were a few concerns that attempts to reduce siting time added to one’s work and cognitive load:

“There’s one more thing to manage and think about.” (P3)

“Standing for eight hours a day doesn’t sound like an achievement, sounds like part of work.” (P1)

That said, participants expressed surprise at how much they liked using the sit-stand desk and the freedom of movement that it gave:

“I have enjoyed using the desk more than I thought I would, and I would be very sad to see that go.” (P13)

“I think just having that freedom to be able to stand or sit has been really nice.” (P8)

A few participants reported dancing at their sit-stand desks:

“I would have my headphones on and I’ll ((laughs)) sometimes be dancing at my desk. Which was quite cool.” (P7)

“It was nice to actually stand and move, and, you know, hop from foot to foot. Or dance.” (P1)

While the intervention was delivered as planned, components of it like the computer prompts, emails and education session/materials may not have been received as intended.

Overall, participants felt the prompt software was acceptable as an awareness tool, but was easy to ignore:

“It [the prompting software] didn’t irritate me. It was fine.” (P12)

“If I get carried away with the emails or with phone calls, the prompt was very helpful in that sense.” (P11)

“Yes, so I would just cancel it [the prompt] and then it would, kind of like, another thing would get in the way, and I’d kind of, I’d just keep going as I was.” (P8)

“So, although it [the prompting software] prompted me to stand some of the time I didn’t because I was in the middle of doing something and I was engrossed in doing it.” (P5)

“I became a little bit blasé about the app [the prompting software], you know? It would ping up and I’d just ignore it and yeah. Yeah, desensitised.” (P5)

The computer prompting software had mixed acceptability. For some, the prompt was helpful because of its simple instructions:

“‘Stand up. Sit down. Take a five-minute break. Take a short walk.’ So, that actually, instead, acted as a, almost as an incentive to do more exercise, in a sense ((laughs)).” (P11)

“It’s [the prompting software’s] like a personal trainer really, isn’t it?” (P2)

It was suggested that more invasive prompts would have been acceptable:

“Maybe a little snippet video of some little exercises […] A little window pop-up. ‘Cos that would irritate you and you would have to do it to stop it [the prompt].” (P5)

“Especially for rest breaks, you need a more direct intervention. Like you can’t use anything. So it [the prompt] forces you away from the desk.” (P9)

Shorter breaks away from the desk prompted by the computer software were more acceptable to participants.

“The shorter breaks were the easiest.” (P8)

The prompts had to be re-activated each day from an internet browser, a task which some found overly cumbersome, forcing them to resort to other techniques to help them remember to stand:

"Because the break would be a break away from the screen, it would be difficult. Unless I'm either walking to a meeting or maybe doing some phone calls on my work phone. So, some work, yes. But it was difficult because you’re not directly a part of the computer.” (P11)

“It was just too confusing to work out something that would work – having to reset them [the computer prompt break times]. And it was just, it just became too much of an issue. So that’s why I broke it down into chunks throughout the day, knowing that I’d be standing for a couple of hours and then sitting for an hour or so, you know?” (P1)

### Practicability

Participants talked about the practical elements of using the intervention to gradually reduce their sitting time over the eight weeks and take frequent breaks from sitting. They were able to follow the instructions clearly, and there were few practical limitations. However, attempts to reach the behavioural target (to replace 2–4 h of sitting with standing and frequent posture changes) varied from person to person, and fluctuated over the course of the study:

“I don’t think I dropped off at all in terms of the way we were instructed. […] Had these sort of step increases in the amount of time we were standing, to not attempt to stand for long periods in the first couple of weeks, but then to gradually increase that. So, it just felt that by the end of it, I felt like I was standing at almost every opportunity to stand, I suppose.” (P4)

“I think I did better in the first week. I was more consistent because I used the Pomodoro [computer prompt] thing and then it became a bit more inconsistent. But I can’t say because sometimes I stood up for a bit longer, so it’s a bit of a blur.” (P3)

Being away from the desk (e.g., at meetings) was the most common reason for not practically being able to engage with the intervention:

“I don’t spend a lot of my day at my desk, so, I was quite limited in the amount of time I could spend standing.” (P4)

“It’s fine having a desk intervention but actually how effective is it if you’re only there for like an hour or two hours out of your day? When you’ve got so many meetings?” (P7)

Others described a sense of relapse when the novelty factor wore off and practically it was more difficult to engage in the intervention when work pressures increased:

“People were interested in the desk initially but […] it faded very quickly. Very quickly.” (P6)

“This is the busiest time of the year so the old habits ((laughs)) kind of creep back in.” (P3)

Participants negatively perceived the loss of desk space when changing desk height (the desk was stationed on top of their normal seated desk) and reported, practically, that the mousepad area could be improved. Still, they would prefer this desk to their standard desk:

“I’d find it [adjusting the desk height] kind of a bit interruptive of the flow of whatever I was doing to then have to move the desk down, sit down, slightly change the layout of the desk.” (P8)

“Quite a small restrictive area for the mouse.” (P4)

“When you’re sitting down or standing up you have to use your mouse at a funny angle, at a different angle. But maybe it’s just the layout of the desk.” (P12)

“It [the sit-stand desk] doesn’t feel as elegant as the whole desks that rise.” (P4)

“Because it’s a U-shape, the space that you get is limited. But I’d rather have a standing desk that was of a lower cost, than no standing desk at all and more space at my desk.” (P7)

The raised desk was a physical barrier to informal conversations with colleagues within the office, which was perceived by some as a hindrance to collaboration and by others as a way to eliminate unwanted distractions. There were mixed feelings about the increased visibility of the computer screen while standing:

“If you’re trying to look at somebody over my screen and talk to them at the same time, I couldn’t see them.” (P1)

“If I'm kind of sat down there’s a lot kind of in my eye line in terms of perhaps people turning round, people asking questions etc. Whereas where I was stood up there wasn’t that kind of a, in my eye line, and it was just kind of like the screen to focus on. So I just found I could concentrate on it better and, kind of, less distractions.” (P8)

“I think because it [the sit-stand desk] raised my desk, my computer screen was like visible for everybody else, I think it could, because of my position in the office. So that kinda wasn’t too great.” (P10)

“No, I didn’t take much notice of that [colleagues seeing my screen] ((laughs)).” (P12)

Participants found the physical mechanics of adjusting the desk height practicable but that overcoming the mental hurdle of adjusting the desk was less so:

“It took seconds and then you’re in a new position.” (P12)

“It is just the initial, yeah, lifting it [the sit-stand desk] up and getting going.” (P6)

Prompts were perceived as a useful practical aspect of the intervention, reminding participants when they lost track of time spent sitting. However, work completion often took priority. The prompt that was delivered through the computer was easy to use. However, they did have to set the prompt each day, which was seen as a limitation:

“If it didn’t have the prompts I’d forget because the tasks that I'm doing, I'm involved in, and the time goes.” (P12)

“So, although it [the computer prompt] prompted me to stand some of the time I didn’t because I was in the middle of doing something and I was engrossed in doing it.” (P5)

“When I initially started, I found that if I wasn’t using Chrome [web browser], I wouldn’t get the prompt. So, I’d remember, let’s say maybe at ten thirty that ‘Oh, I haven’t actually started!’.” (P11)

Prompts encouraged time-dependent use of the desk, with participants expressing individual preferences for performing certain tasks while sitting or standing:

“If I need to do physical paperwork, it’s sitting down then. It was very awkward to do that while standing.” (P1)

“If I'm answering emails or if I'm taking phone calls then it was easier to stand.” (P11)

“Sometimes it’s a little easier to sit while I was typing. […] If you’re doing more sort of mouse work, it’s easier when you’re standing up.” (P2)

“I quite enjoyed typing standing up.” (P3)

“Bits of work that require a lot of intellectual effort, sometimes sitting helps. Standing is my natural in terms of quick tasks, emails, even talking on the phone.” (P4)

“We all joked about there was certain things you couldn’t do standing up. Like someone, ‘I can’t do maths standing up,’ or someone said, ‘I can’t make phone calls, I have to sit down for that’ you know? It was sort of, everyone was different.” (P7)

Although delivered as intended, there were indications that the education component (where participants received brief one-to-one guidance, advice, and an information leaflet) was not received as intended. Participants did not recall much, but those that did offered practical suggestions for improvements:

*“It [the education component] was absolutely fine, yeah. I mean we got a handout and then we went through it with [the researcher], as well. He went through the whole thing with us, so that was useful as well, 'cos he got to explain a lot of what wasn’t clear.” (P10)*

*“I can recall [the researcher] coming in and having a chat about sedentary behaviour and gave us a sheet. […] I think that could have been managed a bit more, you know? What the perceived benefits might be? Why it’s important to actually stick to the recommendations throughout the programme. And perhaps we might not have been quite as click-happy as we were. So, it might have motivated people to participate a little more… fully.” (P5)*

*“I guess if there’s some sort of demonstration and maybe some sort of short film about it [the intervention]?” (P3)*

Email content was also not clearly remembered, but the fact they were sent by line managers was memorable and viewed favourably:

*“I honestly don’t remember what the emails were.” (P10)*

*“I liked the approach [the bi-weekly emails] because it was as though, because it’s coming from your manager, it’s more, it feels as though it’s been approved or authorised.” (P11)*

### Effectiveness

A range of positive impacts of the intervention were reported including musculoskeletal health, fatigue, and concentration benefits.

*“I found it [the sit-stand desk] quite beneficial especially for my back and shoulders. I found that standing up every so often did make a difference.” (P11)*

*“You find yourself drifting off and staring into space and it’s less likely when you’re standing.” (P5)*

*“It [the sit-stand desk] made me feel more awake at the end of the day so… because I wasn’t sitting in my chair for a long time.” (P13)*

Participants realised that there were other ways of working that did not involve sitting down:

*“It’s just not necessary to sit down so much and it’s not healthy. […] In our job especially, if it’s about building relationships, a lot of these things you don’t need to be at the desk, you can go away. So, if I'm speaking with a student, I don’t have to sit at the desk, or yeah, there’s just different ways of doing things.” (P3)*

Participants felt more relaxed and perceived that they moved more at their desk when standing:

*“I just felt more relaxed. More, you know, more active.” (P1)*

*“Standing up, I think you do find yourself moving. Just doing just a little bit more stretching.” (P6)*

Physically switching between sitting and standing helped participants to mentally switch gears between work tasks:

*“I felt less tired, felt more able to… and almost, sometimes, just having that switch would be helpful as well. Just switching gears from standing and sitting. And change of task would help.” (P4)*

*“I felt more productive at work while I was part of that study. While standing I felt, like you just, like being able to focus more.” (P1)*

Participants described the formation of new daily routines around sitting and standing:

*“I have somehow embedded it into my daily routine.” (P11).*

*“I tend now to stand most of the time and then if I get tired, I’ll sit down for a while so that it’s flipped ‘round.” (P5)*

*“I just purely, trying to do it [sitting and standing] in the same amount of time at a time. So, if I was standing for two hours then I know I have to sit for an hour, then stand for two hours and break it up knowing that it was the lunch in the middle as well. And so, I just followed that same pattern.” (P1)*

Participants were optimistic that a healthy working environment could result in increased productivity and fewer sick days:

*“If you can show productivity goes up, then it’s worth investing that money yeah. So, and it’s a win-win then 'cos obviously you can, obviously encourage, promote the healthy thing within the office as well […] I think it creates a positive environment within the office.” (P2)*

*“My feeling would be there would be less injuries less time off from back, you know, back pain, less sick days. It’s that type of thing. Because you know if you’re not sitting correctly, standing correctly, there’s more chance that you’re gonna sustain injury, back injury, strain. Whereas if you got an option to change your position… Perhaps you could analyse the number of sick days taken off or not? That type of thing.” (P5)*

Social influences were strongly perceived to affect decisions to sit or stand. Participants felt supported by their colleagues doing the intervention and by the organisation as a whole, which enhanced its effectiveness:

*“It helped that everybody else was… a few more people were doing it [the intervention], as well, so. It’s quite good to get everybody in the office involved and actually doing it.” (P10)*

*“Colleagues [not in the study] were supportive, they were asking about it.” (P3)*

*“I think definitely people were more interested in what was going on, were interested in the idea of standing more at work and kind of being encouraged to do it.” (P8)*

However, day-to-day decisions to stand and break up sitting were strongly influenced by social norms and not wanting to feel self-conscious.

*“There is the consciousness of every half an hour leaving your desk.” (P6)*

*“If I was doing a particular thing with a particular client and it would be inappropriate to stand.” (P5)*

*“Sometimes you’re aware you’re not doing as much work as you should be and when you’re stood up its like, ‘Oh wait, everybody can see I'm not working.’” (P10)*

That said, having an office partner participating led to supportive comments and facilitation of the behaviour:

*“We would joke about ‘How many times have you stood today?’ and ‘Have you put your Pomodoro [computer prompt] on?’” (P5)*

*“If he [colleague] was standing up, I kind of thought, ‘Oh, I’ll stand up too.’” (P6)*

Because participation was voluntary, most people were intrinsically self-motivated, though external motivation via competition was a suggestion:

*“I loved it [the intervention]. It’s my bag anyway. It’s what I'm interested in.” (P5)*

*“I’m quite active anyway, and really enjoyed the opportunity to be more active at work rather than just sitting all day.” (P7)*

*“Make it a competition or something. Whoever’s stood up the longest gets a little something at the end.” (P10)*

### Affordability

The cost of the desk was an obvious affordability issue. Most participants said they would definitely keep their sit-stand desk if purchased by the organisation.

*“It’s purely about cost. Having the right equipment.” (P4)*

*“It’d be nice to keep the desks, think if the department would actually get some standing desks for everybody, it would be quite beneficial.” (P10)*

However, participants felt that employers would need an incentive such as improved productivity to motivate them to invest in sit-stand desks:

*“If you could associate productivity going up then employers are more in line to actually invest in those [sit-stand desks].” (P2)*

### Side-effects

Back pain resulting from trying to stand for 2–4 h was occasionally reported as a negative side-effect. Participants thus adjusted their standing time to manage this:

*“The initial few weeks were fine. It was in the latter I think, the latter two weeks, for the time standing was longer I found that aggravating the base of my back a bit. Not to an extent I… just that I knew, you know, I’d… the shorter periods of time, more frequently worked better, I felt.” (P12)*

*“Start off with an hour a day and start over the week start increasing it. ‘Cos if somebody jumps in straight away, they’ll probably be wrecked at the end of it.” (P2)*

This was tempered somewhat by several participants reporting that instructions to gradually build up standing time was helpful:

*“It [using the sit-stand desk] was a gradual process. So, you just start increasing the amount of time you’re actually standing. So, it’s, I guess, as a seamless motion through the eight weeks, as such, by the time you get to the eight [weeks].” (P2)*

*“I think it [the eight weeks] was enough of a timeframe to get us used to standing and increasing it.” (P1)*

Moderate to vigorous physical activity may have been negatively impacted:

*“Normally I’d go up and down the stairs. So maybe that means my physical activity is reduced because I’m not going up and down the stairs anymore, so that might be an unintended effect from the study.” (P7)*

### Equity

There was some indication of the negative potential for creating a 'haves' versus 'have nots' work environment:

*“Fundamentally, 'cos they [non-participating colleagues in the office] didn’t have the equipment available to them they couldn’t actually do much at their workstation.” (P8)*

*“I think potentially the negative would be if, for example, you had someone in the office who suffered from back problems, etc., and couldn’t stand and everyone else could, it might create almost like an isolation effect on people.” (P8)*

Balance of power may have affected decisions to stand. Self-consciousness about standing may have been minimal while using one’s personal sit-stand desk, but there were other work situations where doing so would be perceived as challenging authority:

*“Nobody was thinking differently about people who were standing. There was no stigma, or, you know, nobody felt they were exposed.” (P4)*

*“It’s definitely changed my behaviour within the office, and if I didn’t have a standing desk, I think I would still be more conscious about moving.” (P13)*

*“It [the meeting] would have been standing, well if he [my manager] was standing, yeah. Otherwise, it was the fact that I have to stand, and he’s sat down and you can’t just sort of lean over him. So, it’s like, OK, I’ll sit down for this, and I’ll make the time up later.” (P10)*

Participants suggested testimonials would aid recruitment of those most sedentary:

*“I’m certainly not fit but I don’t know […] case studies with people that thought that [they were not confident to use a sit-stand desk] and tried it and actually, ‘OK, […] right, I thought the same as you, and I didn’t think I could do it. But this is what I did and it was absolutely fine.’” (P13)*

## Discussion

Based on assessment in the context of the APEASE criteria in this study, the intervention was found to be highly acceptable to participants (Acceptability). The intervention and BCTs used were well-tailored to the specific workplace and were delivered and mostly received as planned. Full engagement with all aspects of the intervention over the eight-week intervention period varied with individual preferences and workload (Practicability). The intervention had perceived positive impacts on musculoskeletal health, fatigue, concentration levels, and sitting behaviour (Effectiveness). Cost of the desk was cited as a barrier to uptake in the wider organisation and management buy-in was needed (Affordability). The potential to aggravate back pain was the most commonly cited side-effect of the intervention (Side-effects). There was the potential to create a ‘haves' versus 'have-nots’ situation where only those who can afford the desks have the opportunity to receive the health benefits (Equity). However, the intervention may help sedentary workers stand and move more, thereby improving cardiometabolic health.

### Acceptability

The intervention was acceptable and practical for delivery in the workplace, but individual preferences impacted how participants engaged with elements of the intervention. In the current study, computer prompts were perceived as straightforward, easy to follow, and acceptable as an awareness tool. This is consistent with prior research using prompting software to reduce sitting in office workers [12]. Short breaks away from the desk were more acceptable than longer breaks. Overall, sit-stand desks were highly acceptable, in part because they gave users the freedom to choose their working posture, and practicable because participants could actively work from them. Remembering to change posture could sometimes feel like adding to one’s workload. Sit-stand desks have been identified as a key driver of behaviour change [14]. Indeed, sedentary time is largely replaced by standing in multi-component interventions incorporating sit-stand desks [14, 19–22]. The sit-stand desk BCTs used here, ‘Adding objects to the environment’ and ‘Restructuring the physical environment’, frequently appear in promising interventions to reduce workplace sedentary behaviour [52] and improve cardiometabolic risk outcomes [27]. Sit-stand desk thus appear to be an acceptable intervention component for reducing workplace sitting.

Previous research attests to the fact that people have different preferences for standing at sit-stand desks, breaking up sitting time, receiving feedback on behaviour, and how they ‘routinise’ their day [20]. Multi-component interventions offer a way to address multiple levels of influence on behaviour by providing a variety of targeted BCTs, but they may not all be received as intended [53, 54]. In the present study, for example, bi-weekly emails (BCT: Social support [unspecified]) and education materials (BCTs: Instruction on how to perform the behaviour, Credible source, Information about health and emotional consequences), though acceptable, were not well recalled. It may be that the majority of participants did not require these BCTs in order to perform the behaviour, or perhaps the mode of delivery (i.e., text-only emails) did not effectively deliver these BCTs. Future interventions could include a choice of BCTs and/or modes of delivery that allow for a more personalised approach to behaviour change. Participant-tailored behaviour change guidance provided in one sit-stand desk intervention led to large standing time improvements (3 h and 14 min per day) [20]. There are difficulties with this approach, though, in that workers may receive additional and/or differing BCTs, which may affect effectiveness evaluations. This could be tempered somewhat by having a set of core BCTs that all participants receive [20]. The benefits of personalisation may outweigh the disadvantages, namely increased engagement with intervention components and ultimately, improved sitting reductions. Acceptability was high overall for this multi-component intervention, but further personalisation while maintaining a core set of BCTs may lead to improved behavioural changes.

### Practicability

Participants expressed individual preferences for completing certain tasks when standing or sitting, meaning that there were still some barriers for reducing sitting time. Some of these tasks were of the manual sort (e.g., typing or using the mouse), and others were of the intellectual sort (e.g., reading and comprehension). The physical act of typing, for example, was perceived as easier to complete sitting down for some participants. This may be because it requires manual input from a keyboard and sitting provides better ergonomic support for the arm and wrist. Certainly, this was the case for mouse pad use when standing as participants overwhelmingly described having to position their wrist at a ‘funny angle’. Desk-mounted sit-stand workstations have been identified as a potential barrier to standing when work tasks require desk space [55]. To increase the effectiveness of sedentary workplace interventions, addressing the remaining barriers to sitting less is key. Mounted sit-stand desk design improvements, such as a larger mouse pad area, may overcome such issues. Additionally, future studies could consider human–machine interface technologies (e.g., dictation software, trackpads) that would lessen the burden of manual input issues like typing and mouse use, which may in turn help those individuals who find this a barrier to achieving sitting time recommendations.

### Effectiveness

Effectiveness of an intervention is largely dependent on whether it is delivered and received as designed via the means intended [53]. Participants confirmed that computer prompts (BCT: prompts/cues), for example, were largely delivered as intended and useful for generating an awareness of their sedentary behaviour at work. It has been suggested that the first step in reducing sedentary behaviour may be to bring sitting into conscious awareness [56]. Participants talked about clicking away the computer prompt during high workloads. Furthermore, participants in the study described falling back on old habits of sitting when work demands increased, and that this fluctuated over time. Workload is commonly cited in the literature as a barrier to sitting reduction during interventions [17, 20, 23, 57]. Sitting is generally perceived as a necessary means for completing one’s work, which may inadvertently reinforce it [56]. Prompt software has been shown to reduce device-assessed mean sitting time by ~ 12–14 min per eight-hour workday [58, 59] and by 26–47 min per day (depending on break time/frequency) [60]. Participants suggested the use of more invasive and engaging computer prompts to overcome the competing motivation to sit. Exertime®, for example, is a computer programme that locks the user out of their computer screen every 45 min with instructions to complete 1–2 min of light activity like standing, walking, stretching or office exercises like chair squats [61–63]. However, the invasive nature of the prompts was perceived unfavourably and seen as disruptive to participants’ workflow in a feasibility trial [59]. Future research should explore ways that interventions can motivate participants to overcome the competing attraction of work task completion. This could be achieved potentially by including strategies to self-monitor behaviour [64] and/or providing non-specific rewards, as suggested by some participants in the present study.

Two important considerations of workplace interventions are sustainability and maintenance of the behaviour over time. Along with new routines and habits, this study evidenced some cultural shifts that may be occurring in the workplace. Participants spoke about wanting to incorporate standing into more of their day and not just at their desk, but at other times like during meetings. This change may have been facilitated by the social support seen and felt by participants from co-workers within their office cluster and the wider organisation (e.g., the bi-weekly emails from management). Earlier research points to a ‘culture of expectation’ where workers who are not sitting are perceived to not be working [65]. Workplace culture, defined as an organisation’s values, underlying assumptions, and behaviour, has been shown to influence sitting time [66]. However, the current study has shown that a multi-component intervention that simultaneously targets the individual, the organisation, and the environment, can begin to influence this culture. Future interventions should, therefore, employ a multi-component approach to continue to promote a less sedentary workplace culture for sustainable behaviour change.

### Affordability and Equity

Affordability of the sit-stand desk for individual workers was identified as a barrier, something which could lead to a ‘haves’ versus ‘have nots’ situation, potentially exacerbating health disparities. Participants reported that they would like to keep the sit-stand desks if their organisation purchased them for reasons such as improvements in concentration and muscle fatigue. However, they felt management buy-in was unlikely without clear productivity benefits. Research suggests that there is little detriment to work performance with active workstations [67] and a large multi-component intervention incorporating sit-stand desks found an improvement in job performance, work engagement, occupational fatigue and sickness presenteeism [68]. Furthermore, a preliminary cost–benefit analysis showed that a multi-component sit-stand desk intervention may be a good business investment with a 256% return on investment (£2.56 returned for every £1 spent), based on productivity loss due to poor health averaged over 12 months [69]. Sit-stand desk interventions (with or without counselling) also have a high probability of being cost-effective in the primary prevention of cardiovascular disease in office workers [70]. Due to the inherent affordability issues with sit-stand desks, it would be better if the organisation was the provider, rather than it being down to individual employees to purchase their own, so there would be equitable access. Future interventions should address management misconceptions around intervention costs versus benefits in order to improve organisational buy-in. At the same time, intervention research should identify the most equitable solutions to reducing workplace sitting time, with or without the use of sit-stand desks. In a whole systems approach, governing bodies should consider policy categories (e.g., guidelines, service provision, fiscal measures) for educating, training and incentivising organisations to exploit the public health benefits of sedentary behaviour change [28, 29]. Sit-stand desks, along with training and prompting software, were feasible in the workplace, but to reduce the potential for health disparities linked to affordability, organisations need to provide fair and equal access to behaviour change resources for all of their desk-based workers.

### Side-effects

The were some reports of back pain during the intervention, but this was managed by gradually building up to standing for longer periods and by participants changing postures more frequently. Intervention participants often perceive sit-stand desks as providing musculoskeletal pain relief from excessive sitting [71, 72]. Indeed, a number of participants in the current study experienced beneficial effects in terms of their musculoskeletal health (feeling relaxed, reduced shoulder pain), as well as feeling more awake and experiencing improved concentration. One previous study found the odds of experiencing back pain to a level impacting normal activities was less for those in a multi-component sit-stand desk intervention compared to a usual-routine control group in a 12 month follow-up [68]. One expert statement recommends that prolonged static posture (sitting or standing) should be avoided [7]. However, clear, evidence-based guidelines around workplace sitting time reduction and musculoskeletal health are still needed [73]. Highlighting the potential for back pain and disseminating strategies on how to prevent it at the start of an intervention, such as educating participants on how to pay attention to body discomfort as a cue to change posture, may be a feasible solution [20]. Also of note in the present study, standing at the sit-stand desk replaced at least one participant’s stair walking breaks. Others reported dancing at their desk, something which they did not do prior to the intervention. Thus, additional behavioural support should be considered in future interventions to help maintain (or even increase) workplace physical activity levels. Musculoskeletal discomfort should also be monitored, and interventions adjusted for individual participants if symptoms occur.

### Strengths and limitations

A strength of this study was a systematic approach using the APEASE criteria to understand intervention acceptability and feasibility. Qualitative studies of sedentary behaviour in the past have largely used thematic analysis [20, 22, 57] or grounded theory [16] for understanding engagement with intervention resources. Inductive thematic analysis allows for a rich, detailed, and nuanced presentation of information [74, 75]. However, the process may not be as transparent in terms of assumptions made during analysis and how the analysis was carried out [75]. A further strength of the present study was the mapping of the APEASE framework to identified themes to aid transparency and rigor to the feasibility analysis. Interviews were conducted with a highly educated, predominantly female, university staff sample which may limit the generalisability of the findings. These demographics are typical of other post-intervention qualitative studies and thus extends knowledge in this field [17, 20, 23, 57]. Additionally, interviewees were characteristically representative of the intervention group as a whole [34]. Interviews with both managers and non-managers in this study advances a more comprehensive understanding of the feasibility and acceptability of sedentary interventions in the workplace. It is worth noting, however, that due to the voluntary nature of the present study, the perceptions expressed by participants may not be wholly representative of those who did not take part [71]. Furthermore, views expressed are perceptions only and may not reflect actual behavioural changes [71].

## Conclusions

This theory-derived, multi-component sit-stand desk intervention was highly acceptable to office workers, safe to deploy, and useful in reducing and breaking up sedentary time at work. Prompting software and sit-stand desks were effective at reducing sitting time, but additional BCTs like behavioural self-monitoring and/or non-specific rewards, for example, may be needed to induce and motivate participants to achieve sitting reduction levels in line with public health targets. Employees reported positive impacts of reducing sitting on concentration levels and appreciated having the choice to work sitting or standing at will. Affordability remains an issue with management buy-in and is likely to be tempered by perceived cost-benefits. Personal preference played a substantial role in how participants engaged with intervention components, demonstrating a need for more personalised approaches to sedentary behaviour interventions while maintaining a core set of intervention BCTs. This research has extended knowledge in the area of sedentary workplace interventions, and offers a novel application of the APEASE criteria to aid future intervention development.

## Supplementary Information

**Additional file 1. **Table that shows the completed COREQ checklist.xlsx.

**Additional file 2.** Table that shows the interview schedule.pdf.

## Data Availability

The qualitative datasets generated and/or analysed during the current study are available from the corresponding author upon reasonable request.
