# Supplementary material for: Evaluating a multi-component intervention to reduce and break up office workers’ sitting with sit-stand desks using the APEASE criteria
Source: BMC Public Health. 2022 Mar 7;22:458. doi: 10.1186/s12889-022-12794-w (PMC8902706; doi:10.1186/s12889-022-12794-w)
Supplement: Supplementary file 2 — Additional file 2. Table that shows the interview schedule.pdf. [file 12889_2022_12794_MOESM2_ESM.docx]

Additional File 2. Interview schedule designed to elicit perceptions of acceptability, practicability, and impacts of a multi-component sedentary workplace intervention in full-time, adult employees.

| Topic | Questions |
| --- | --- |
| **Welcome script** | Thanks for your time in agreeing to speak with me today. I am interested in hearing your views on sitting at work and the intervention that you have recently been involved with. Your answers will help us to design a future workplace intervention. When I refer to ‘the intervention’ I mean the sit-stand desks, the email newsletters, the education session at the beginning and the computer prompts that you received from Samson.  The interview will be recorded, and will take about 25mins to an hour. I may use quotes from our conversation but they will be kept anonymous and I will provide only a pseudonym as a descriptor. I was hoping to take a few notes as we go along – is that ok?  Please know that you are free to stop at any time should you wish to discontinue the interview. I hope you will find it interesting. Are you happy for us to proceed? |
| **Introductory questions**:  Job Role & background | Tell me about your job role and day-to-day tasks.  *Prompt: Describe the layout of the office and your desk within it.*  *Prompt: How many hours a day did you sit prior to the intervention?* |
|  | What motivated you to participate in the intervention? |
|  | Tell me your overall impressions of the intervention. |
| **Intervention experience:**  Use of intervention resources, Facilitators, Barriers | Would you please describe in your own words what the intervention was?  *Prompt: How do you feel the intervention worked? (If they did not feel it worked, how was it supposed to work?)* |
|  | How did you use the desk?  *Prompt: Was your use of the desk time dependent or task dependent?*  *Prompt: What types of tasks did you generally do standing up/sitting down?*  *Prompt: What helped you to stand as much as you did?*  *Prompt: What may have stopped you from standing more than you did? (Footwear, others, office layout)* |
|  | How supported did you feel during the intervention?  *Prompt: By intervention deliverers, by management, by your peers, by technology, by the environment, educational resources?* |
|  | How did your motivation change over the course of the study? |
|  | Overall, what worked well, and why? |
|  | What, if anything, would you change, and why? |
| **Intervention experience:**  Motivation and sustainability [1] | How many people did you notice standing in your office?  *Prompt: What reasons might people give for why they did not use their desk to stand?* |
|  | What strategies might motivate others at work to engage with an intervention like this? |
|  | Which intervention components or habits (if any) do you plan to continue with?  *Prompt: On a scale of 1-10, how confident are you that you can maintain this? (1=extremely unconfident, 10=extremely confident)* |
|  | What would make these changes sustainable in the long term? |
|  | What might get in the way? |
| **Overall thoughts:** Barriers, Facilitators | In general, what is the greatest barrier in implementing an intervention like this in the workplace? |
|  | What would enable this type of intervention to work well?  Prompt: (Education, environment, technology, social support, organizational support, policies, legislation?) |
|  | Would you recommend this intervention to others?  Why is this… |
| **Intervention experience:**  Knowledge, identity, and culture | What is your understanding of workplace sedentary behaviour? How would you describe this to others? What words would you use? [2] |
|  | What have you learned about prolonged sitting and health? |
|  | What is your understanding of someone who is sedentary versus someone who is inactive? |
|  | How has the workplace culture changed as a result of the intervention? |
|  | How do you feel your value as an employee has changed due to this intervention? [1] |
| **Impacts:** Culture, Habits, Health, Productivity | Describe the impacts of the intervention on your:  -Behaviour  What about your…  -Musculoskeletal health  -Physical well-being (illness, absences from work)  -Mental well-being (mood, stress levels)  -Alertness/concentration  -Physical activity and sitting outside the workplace [2]  -Work productivity [1]  *Prompt: Communication*  *Prompt: Collaboration*  *Prompt: Timeliness of task completion/work flow*  *Prompt: Audio/Visual privacy disturbances?* |
| **Intervention experience:**  Monitoring | How were you monitored for change? (Any: Behavioural, musculoskeletal health, physical health, mental well-being, work productivity) |
|  | How did you find the tools/devices used by the researcher in monitoring your behavior? |
|  | What self-monitoring techniques (if any) did you employ? |
| **Closing question** | What else, if anything, do you think we should consider when developing a future intervention to break up sitting time at work? |
|  | In closing, is there anything else you’d like to say about your experience of the intervention, use of the desk, or of wearing the activity monitor? |
| **Debrief script** | I appreciate your time today in taking part in this interview on sitting in the workplace. Once my study is complete the results will be available – you may email me for the details. Also, feel free to get in contact if you have any further questions. Thanks again for your time. |
| **References** | 1. Hadgraft NT, Willenberg L, LaMontagne AD, Malkoski K, Dunstan DW, Healy GN, et al. Reducing occupational sitting: workers’ perspectives on participation in a multi- component intervention. Int J Behav Nutr Phys Act. 2017;14:73.  2. Grunseit AC, Chau JY, van der Ploeg HP, Bauman A. “Thinking on your feet”: a qualitative evaluation of sit-stand desks in an Australian workplace. BMC Public Health. 2013;13:365. |

| Evaluating a multi-component intervention to reduce and break up office workers’ sitting with sit-stand desks using the APEASE criteria |
| --- |
| *BMC Public Health* |
| Marsha L. Brierley, Brunel University London |
| Lindsey R. Smith, University of Bedfordshire |
| Daniel P. Bailey, Brunel University London |
| Samson O. Ojo, University of Bedfordshire |
| David J. Hewson, University of Bedfordshire |
| Sofie A. Every, Brunel University London |
| Taylor A. Staines, University of Bedfordshire |
| Angel M. Chater, University of Bedfordshire |
| Corresponding author: Angel M. Chater, University of Bedfordshire, angel.chater@beds.ac.uk |
